# Mercurials in Dysentery

**Published:** 1852-09

**Authors:** S. B. Hunt

**Affiliations:** Mendon, Monroe Co., N. Y.


### ART. V.—*Mercurials in Dysentery.*

By S. B. Hunt, M. D., Mendon, Monroe Co., N. Y.

In the *Journal* of September last, is an article on dysentery, from the pen of Dr. Morse, of Tuscarora, Livingston Co., followed by some editorial comments. In the following number the eclectic department furnishes the opinions of Dr. Parrish of New Jersey, on the same subject.

It occurred to me at the time, in reading those articles, that they were (especially the first named, with the comments following) rather heterodox in sentiment, departing somewhat from the old landmarks, and discordant with my own (in this disease) somewhat varied experience. I treated, during the months of July, August and September of the year 1848, one hundred and thirty cases of dysentery, occurring in the town of Portage, Livingston Co., in the Cashequa Valley, some eight or ten miles above Tuscarora. In that region, which is annu y devastated by this disease, the epidemic of 1848 will long be remembered.

In the summers of 1846–'47–'49 and '50, in that region, and to a less extent for the past two seasons in this my present location, I had also ample opportunities for testing the various methods of treatment, and arrived at the same general conclusions taught me by the epidemic of 1848. This epidemic, commencing about the 20th July, continued till the close of September. During that time, sixteen deaths occurred of the disease in the township, six of them in my practice. I have full and accurate minutes of the cases as they occurred. I have no intention of afflicting you with a full account of them, but shall rather state the conclusions at which I arrived at the time, with a few deductions forced upon my mind by the fatal cases. After this prelude, you will guess that I am about to reassert the time-honored fame of calomel in this disease, and moreover, to place myself for the nonce, in the ranks of the Hepatic Pathologists, a much abused class of "old hunkers," carrying their conservatism a trifle too far perhaps, yet still possessing many of those sterling attributes of veneration for the past—a sturdy determination to "*hold fast* to that which is good," and who show a degree of energy in attacking a disease, shocking to the sensibilities of all homeopathists, and their cousins, the expectants.

In taking the epidemic of 1848 as my theme, I do so because it is more convenient, and presents types of all the modifications of dysentery I ever met. I lay down the following propositions to start with.

1st. It was malarial in its origin.

2d. There were unmistakable signs of hepatic disturbance preceding the disease, sometimes by many weeks.

3d. That it was *not* purely inflammatory in its character.

1st. As to its malarial origin. The cases occurred principally upon the borders of the Cashequa Creek, which, having a rapid fall, was interrupted in its course by several mill ponds, which were frequently drawn off and slowly filled again, leaving a mass of decomposing vegetable matter, exposed to the rays of a hot sun. Other cases occurred at the Deep Cut of the Genesee Valley Canal, a high and breezy location, but where men, women, and children, pigs and poultry, lived huddled together in miserable shanties, and where vast quantities of earth were daily turned up to the sun. Other cases occurred on the road leading south from the Deep Cut, in a location apparently healthy, but really exposed to the malaria brought over it by westerly winds, from a large swamp a short distance west. Lying to the west of the town, and forming its boundary, is the Genesee river, with wide flats or bottoms pregnant with malaria, and where intermittents and dysentery are household names.

2ndly. Signs of Hepatic Disturbance.—Every one who has seen a case of dysentery, recollects the parti colored stools so indicative of "*bilious*" disturbance. [I have still left moral courage enough to say sometimes to a patient, "*You are bilious, Sir!*"] But here the objection of Doct. Parrish meets us, this is a sympathetic derangement depending upon, and cured with the inflammation below. It would seem that portal congestion, setting back the blood upon the capillaries of the rectum, would be quite as likely to cause engorgement and inflammation there, as the *vice versa* process would be to cause portal congestion; but, dropping this argument, let us look at the premonitory symptoms of dysentery.

I extract from my note book:—"This class of cases was indicated by symptoms of hepatic disturbance, *preceding sometimes by weeks* the onset of the disease. Yellowness of skin and conjunctiva, sleepiness at noonday, bitter taste in the fauces, pain in the right shoulder, with uneasiness in the hypochondrium, and consequent disposition to lie on the right side, were generally present."

The third proposition that it was not purely inflammatory in its character, follows naturally the other statements, and requires little proof. Many cases were essentially typhoid in their character, and the patients were prostrated at the onset of the disease to such a degree as no purely inflammatory disease manifests.

And now I wish to put in a word or two lest I may be misunderstood as to the treatment of dysentery. In the class of cases which I have been describing, and which I believe make up a large modicum of the cases coming under treatment, calomel was the sheet anchor. Given, combined with sufficient opium to control its cathartic tendencies, repeated at intervals of three or four hours, and continued till bilious stools followed its exhibition, it was followed with the happiest results. In cases where the liver did not seem to be implicated, a favorite plan of treatment was to give at night ten grains pulv. Dover, repeated in two hours should the first dose fail to quiet the tormina. This was followed in the morning by ℥ii sal Rochelle, given in light mutton broth. Frequently this was sufficient for a cure; if not, the plan was repeated. In other cases, a full dose of opium, (3 or 4 gr.) cut short the disease.

One word about the use of sal Rochelle. It is less nauseous, and more reliable than castor oil, and much more rapid in its action, which is a very desirable quality. I have never seen any untoward symptoms of irritation following its use, and its stimulant effect upon the duodenum, assists in rousing the liver.

As to astringents, I have given the whole round of them a more thorough trial than I shall ever venture upon again. I do not know but Dr. Condie can check the discharges with his pulv. acet. plumbi ipecac. et opii, but I cannot. I have failed entirely a hundred times in trying astringents, and so wasted valuable time.

As to the use of cathartics, I do not think that any person can prescribe a set rule for their administration. I am partial to them, but my rule is to wait until I find that the contents of the bowel are trickling down over the inflamed surface, and then to give the sal Rochelle, sweep out the bowels thoroughly, and then close up with opiates, or should the hepatic symptoms be present, with calomel and opium, until the indication for cathartics comes round again.

My note book furnishes me another text, and a solemn one to preach from, as to the use of mercurials. I will give merely an abstract, as I have already said more than I intended.

Two cases of dysentery occurred in children of the same age, in houses a quarter of a mile distant, but exposed to the same miasmatic causes. In each case, my little patients had careful nursing. In the one, I suspended mercurials on the evening of the 28th August, on account of the apparently moribund condition of my patient. But he lived, and bore the most exhausting discharges for four days thereafter, when he died. The other case had symptoms no less severe. He, too, was moribund; but I followed him up with calomel to death's door, and when, not expecting to find him alive, I visited him, and gave him a large injection of cold water, which was followed by a copious forced evacuation, he convalesced rapidly. The added experience of four years has given me no reason to change the opinion expressed in my note book at the time, and which I append as a clincher to my argument. I quote—" In this case, the use of mercurials was continued much longer, and through greater debility than in the first; and however disagreeable it may be to acknowledge faults in a fatal case, yet the conclusion forces itself upon me, that had the use of calomel been continued through the night of the 28th, in the first case, a different result would have been obtained. I believe it to be good practice in this form of dysentery, to continue mercurials until approaching death, if death must come."

MENDON, *July* 19, 1852.

